# Discovery of PilU as a second type IV pilus retraction motor in *Myxococcus xanthus*

**DOI:** 10.1128/jb.00248-26

**Published:** 2026-08-12

**Authors:** Mahdia Rahman, Kalpana Subedi, Andrea Harms, Daniel Wall, Anke Treuner-Lange

**Affiliations:** 1 Department of Molecular Biology, University of Wyoming 4416, Laramie, Wyoming, USA; 2 Max Planck Institute for Terrestrial Microbiology 28310, Marburg, Germany; Dartmouth College Geisel School of Medicine, Hanover, New Hampshire, USA

**Keywords:** myxobacteria, type IV pili, twitching motility, social motility, pilus retraction

## Abstract

Type IV pili (T4P) drive social (S) motility in *Myxococcus xanthus* through cycles of extension and retraction powered by the ATPases PilB and PilT. Although the canonical retraction ATPase PilT is essential for force generation, *M. xanthus* encodes four PilT-like paralogs whose contributions to motility remain unclear. Here, we identify MXAN_1995 as the long-sought PilU protein that serves as a second T4P retraction motor. A frameshift mutation or deletion of *pilU* abolishes S-motility while preserving pilus assembly and exopolysaccharide (EPS) production, phenocopying the *pilT* mutant. Single-cell analyses revealed that Δ*pilU* mutants exhibit rare, low-force movements, consistent with a role for PilU in force generation. Fluorescence microscopy showed that PilU localizes predominantly to cell poles, similar to PilT, and that PilU localization is independent of PilT but partly dependent on core T4P assembly proteins. Notably, calcium differentially modulates motility, enhancing movement in wild-type cells while suppressing motility in Δ*pilU* mutants, indicating a role for PilU under varying environmental conditions. Structural modeling, together with an intragenic suppressor, suggests a regulatory function for the intrinsically disordered C-terminal region of PilU. As found in other bacterial species, our findings establish PilU as a secondary retraction ATPase and uncover a dual-motor retraction system that is environmentally responsive and mechanically tunable in *M. xanthus*.

**IMPORTANCE:**

T4P are widespread motility and adhesion systems that enable bacteria to move, interact, and form multicellular communities. While the primary retraction ATPase PilT is well characterized, the function of additional PilT-like proteins remains unclear in many species. This work provides the first mechanistic characterization of PilU in *Myxococcus xanthus*, a model for multicellular behavior and T4P biology. We show that PilU is essential for productive T4P retraction, functioning as an accessory motor that enhances or stabilizes PilT-driven force generation. We further reveal that PilU activity is modulated by environmental calcium and depends on a flexible C-terminal region that influences motor dynamics. These findings uncover a dual-motor architecture that enables adaptive control of T4P retraction in response to environmental cues.

## INTRODUCTION

Motility is a key determinant of bacterial fitness, contributing to habitat colonization, virulence, and biofilm formation (1). One widespread mechanism of bacterial motility is mediated by type IV pili (T4P), dynamic cell-surface filaments that also participate in pathogenicity, DNA uptake, protein secretion, and biofilm development. T4P-driven movement results from repeated cycles of pilus extension, surface attachment, and retraction, generating retraction forces exceeding 100 pN and making T4P among the most powerful biological nanomachines described (2–4).

*Myxococcus xanthus* is a gram-negative, soil-dwelling bacterium renowned for complex multicellular behaviors, such as predation, development, and sporulation, all of which rely on coordinated motility (5). This organism employs two genetically and mechanistically distinct motility systems. Adventurous (A) motility supports single-cell movement on firm surfaces and depends on Agl/Glt focal adhesion complexes spanning the cell envelope (6). In contrast, social (S) motility predominates on moist surfaces and is powered by T4P. Secreted polysaccharides referred to as exopolysaccharides (EPS) are important for S-motility and can affect T4P extension or retraction (7). Specifically, EPS serves as a binding substrate for pili, enabling productive traction and cell movement. Consequently, mutations that disrupt EPS production or secretion result in severe defects in S-motility despite the presence of pili, demonstrating the critical role of EPS in T4P-dependent locomotion.

The extension and retraction of T4P are driven by the conserved T4 pilus machinery (T4PM) (8). In gram-negative bacteria, this complex spans the outer membrane, periplasm, inner membrane, and cytoplasm and comprises approximately 15 conserved components (9, 10). The machine consists of a basal body embedded in the cell envelope and an external pilus fiber composed primarily of the major pilin PilA. Basal body subcomplexes include the outer membrane pore, alignment complex, and motor complex. In addition, a priming complex containing PilY1 and several minor pilins facilitates pilus assembly and mediates surface attachment and retraction signaling (9).

T4P dynamics are powered by the motor complex, which includes the platform protein PilC and the ATPases PilB and PilT. PilB and PilT associate with the cytoplasmic base of the T4PM in a mutually exclusive fashion to power T4P extension and retraction, respectively (10). PilB and PilT belong to the ASCE family of AAA^+^ ATPases and assemble as hexameric rings surrounding a central pore (8, 11–14). Through interactions with PilC and PilM, ATP binding and hydrolysis induce coordinated conformational changes within the hexamer that are thought to drive PilC rotation and pilus polymerization or depolymerization (15–18).

The rod-shaped *M. xanthus* cells move across surfaces in the direction of their long axis, exhibiting distinct leading and lagging cell poles. While the T4PM core is present at both poles, T4P only assembles at one pole at a time, allowing unidirectional movement with a piliated leading and a non-piliated lagging cell pole (5, 19). Consistent with the unipolar T4P formation, the PilB extension ATPase localizes to the leading cell pole, while PilT predominantly localizes in a bipolar asymmetric pattern (20).

The retraction ATPase PilT functions as the primary depolymerization motor and is required for high-force, high-frequency pilus retraction (3, 4). Loss of *pilT* in many bacteria abolishes twitching motility, DNA uptake, and surface-associated behaviors, frequently resulting in hyperpiliation due to continued extension in the absence of retraction (3, 4, 8, 21–23). In several gram-negative bacteria, including *Vibrio cholerae*, *Pseudomonas aeruginosa*, and *Neisseria* spp., a second PilT-like ATPase, PilU, is encoded immediately downstream of *pilT* (24–26). PilU shares substantial sequence homology with PilT and exhibits ATPase activity *in vitro*, yet its contribution to motility differs across systems. In some organisms, PilU functions as an accessory or conditional retraction motor, enhancing retraction efficiency under high-load conditions or contributing to sustained force generation (3, 25, 27, 28). In other systems, PilU activity is PilT-dependent, suggesting a model in which PilU modulates retraction dynamics rather than acting as an independent motor (24, 25). These observations support a division of labor among retraction ATPases, with PilT providing high-force retraction and PilU fine-tuning retraction efficiency, processivity, or coordination with environmental cues.

Notably, *M. xanthus* encodes an expanded set of PilT-like AAA^+^ ATPases, including four paralogs in addition to the canonical PilT, with sequence identities ranging from 58% to 66%. This expansion suggests increased regulatory complexity and potential specialization of retraction motors in a bacterium that relies heavily on collective motility. T4P retraction normally generates sufficient force to pull cells forward, with wild-type cells producing ~150 pN of stall force, yet *pilT* mutants can still generate high-velocity retractions with lower force (~70 pN), indicating the existence of an additional retraction motor in *M. xanthus* (3, 29). Here, we identify MXAN_1995 as the long-sought PilU retraction ATPase involved in T4P-dependent S-motility. A frameshift mutation or deletion of MXAN_1995 abolishes S-motility without affecting pilus assembly or EPS production, thus phenocopying a *pilT* mutant. Fluorescence microscopy further reveals that the protein localizes to the cell poles and colocalizes with PilT. Together, these findings establish *MXAN_1995* as the *M. xanthus pilU* gene and support a model in which multiple retraction ATPases cooperate to modulate force generation and pilus dynamics during S-motility.

## RESULTS

### Identification of *MXAN_1995* as the *pilU* gene required for S-motility

To comprehensively identify genetic factors involved in T4P function in *M. xanthus*, we have characterized an extensive collection of historic unbiased S-motility mutants generated by chemical and UV mutagenesis in strains that lacked A-motility in the Kaiser laboratory (12, 30). Sixty of these mutants were previously analyzed by transmission electron microscopy and quantified for pili production (14). Using this approach, the *pilT* and *dsp*/*dif* genes, responsible for EPS production, were identified, where mutations in these genes resulted in S-motility defects, although pili were still assembled. Additional S-motility mutations mapped outside of the *pilT* and *dsp*/*dif* loci that still produced pili and EPS, which interested us because they imply a role in pili function rather than pili biogenesis. Two of these historic S-motility genes, *sglS* and *sglT*, we recently showed had redundant essential roles in T4P function at high temperatures (31, 32). Here, we investigate an additional S-motility mutation in the historic DK2120 strain, which contained the *cglB2* (adhesin protein) A-motility mutation (33, 34). The DK2120 S-motility mutant exhibited a *pilT*-like phenotype, producing pili and forming cell clumps during liquid growth, but also displaying a temperature-sensitive (Ts^−^) S-motility phenotype, meaning it was motile at lower temperatures (16°C) but non-motile at higher temperatures (25–33°C) (14).

**Fig 1:**
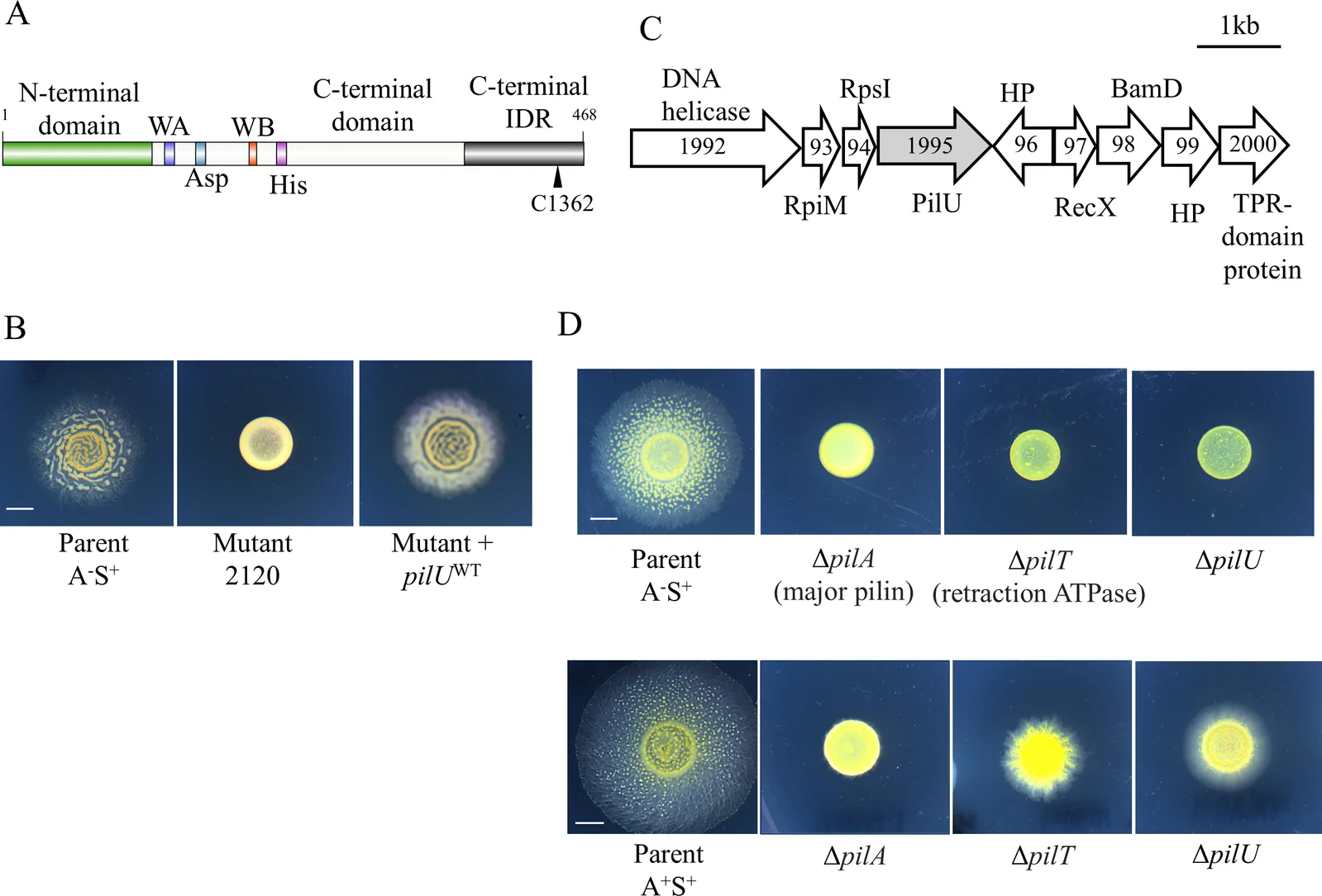
Complementation and genetic context of *pilU*. (**A**) Domain organization of PilU. Vertical bars indicate four conserved motifs in the ATPase domains: Walker A (WA) box, Asp box, Walker B box (WB), and His box. Black triangle marks the frameshift mutation in DK2120. (**B**) Complementation of the DK2120 *pilU-fs* mutant with WT *pilU*. Parent strain DK1218. (**C**) Schematic of the genomic region around *pilU*. Numbers represent MXAN locus tags, while arrows indicate gene orientations. Predicted gene products indicated above or below arrows. HP, hypothetical protein; DNA helicase, ATP-dependent DNA helicase; RplM, large subunit ribosomal protein L13; RpsI, small subunit ribosomal protein S9; RecX, regulatory protein; BamD, outer membrane protein assembly factor; TPR-domain protein, tetratricopeptide repeat domain-containing protein. (**D**) Phenotypic analysis of *pilU* deletion mutants in A^−^S^+^ (parent strain DK1217) and A^+^S^+^ (parent strain DK1622) backgrounds with Δ*pilA* and Δ*pilT* controls. All S-motility assays were performed on soft agar with 2 mM CaCl_2_; scale bars: 2.5 mm.

To identify the DK2120 S-motility mutation, its genome was sequenced. A single cytosine insertion mutation within a homopolymeric tract of five cytosines near the 3′ end of *MXAN_1995* caught our interest because this ORF was a *pilT* paralog (Fig. 1A). To test our hypothesis that the *MXAN_1995* frameshift mutation caused the S-motility defect, the wild-type (WT) gene was cloned in pMR3487 and integrated into the DK2120 genome. Complementation tests demonstrated that the WT *MXAN_1995* allele restored S-motility (Fig. 1B), confirming that the *MXAN_1995* mutation was responsible for the S-motility defect and, hereafter, was called *pilU*. Comparative genomics found that *pilU* was typically located within a conserved four-gene cluster in the Myxococcales phylum (Fig. 1C), and in *Archangium* and *Melittangium*, it was found in proximity to *pilY1* and genes encoding minor pilins (Fig. S1). Sequence analysis showed that PilU contains the conserved Walker A and Walker B motifs, as well as the Asp and His Boxes characteristic of P-loop NTPases involved in ATP binding and hydrolysis (8, 35). In addition, PilU possesses a predicted C-terminal ~112-amino acid intrinsically disordered region (IDR) (Fig. 1A) that is only conserved in myxobacteria.

To independently validate the functional role of *pilU* and its null phenotype, we generated an in-frame deletion in an A^−^S^+^ background (DK1217; *aglQ1*). The resulting Δ*pilU* mutant showed a non-motile phenotype at 33°C that was indistinguishable from Δ*pilA* and Δ*pilT* mutants (Fig. 1D, upper panel), consistent with a loss of T4P-dependent motility. However, this Δ*pilU* mutant exhibited a Ts^−^ motility phenotype similar to DK2120 (*cglB2 pilU-fs*), both showing residual motility at lower temperatures (18°C) (Fig. S2). Deletion of *pilU* in an otherwise WT A^+^S^+^ background (DK1622) resulted in a severe S-motility defect at 33°C, characterized by limited flares of cells emerging from the colony and markedly reduced swarming on soft (0.5%) agar (Fig. 1D, lower panel). The residual colony expansion, which contrasted with a Δ*pilA* mutant, suggested that the Δ*pilU* mutation, in the presence of A-motility, retains limited soft agar colony expansion (swarming). This phenotype was similar to that of the Δ*pilT* mutant (Fig. 1D), in which we attribute soft agar swarming to A-motility when cells can bundle together through pili and EPS production, thereby facilitating soft agar swarming (14, 36). Together, these results demonstrate that *pilU* was an important component for S-motility and likely functions with the T4P retraction machinery in *M. xanthus*.

### PilU functions as a PilT-like retraction motor

**Fig 2:**
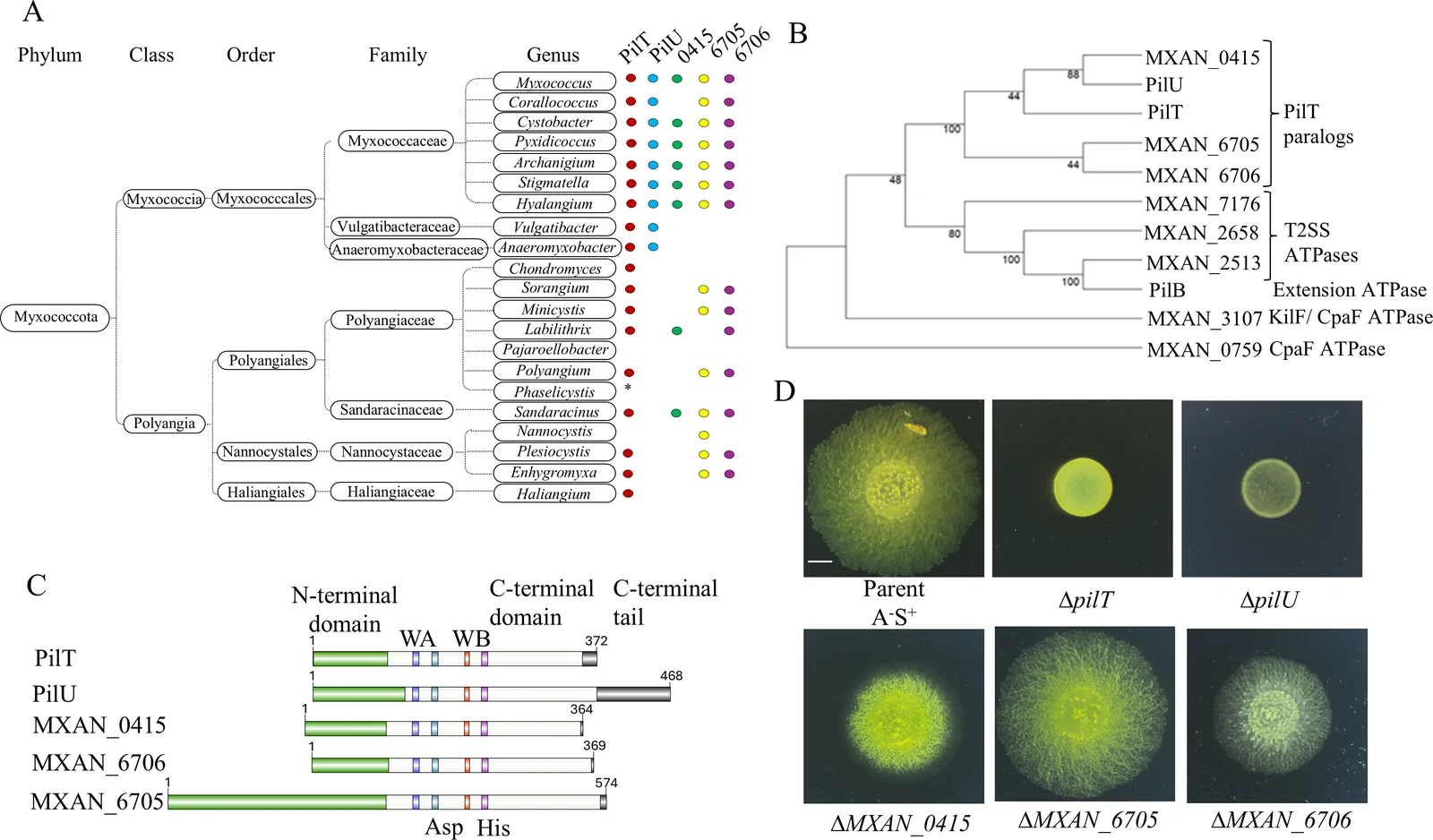
Distribution and phylogeny of PilT paralogs in Myxococcota. (**A**) Occurrence of *pilT* (*MXAN_5787*), *pilU* (*MXAN_1995*), and three additional *pilT* paralogs across phylum Myxococcota. The presence of paralogs is indicated by colored circles (E-value < 10^−10^). Blank, not present; asterisks, genomes unavailable. (**B**) Phylogenetic relationships among hexameric ATPase family in *M. xanthus*. (**C**) Domain organization of PilT paralogs. The N- and C-termini are conserved, except that *MXAN_6705* and *pilU* have longer N- and C-termini, respectively, which are predicted to be intrinsically disordered. Vertical bars indicate four conserved sequence motifs in the ATPase domains: Walker A (WA) box, Asp box, Walker B box (WB), and His box. See Fig. S1 for sequence alignment. (**D**) Swarm phenotypes on soft agar with 2 mM CaCl_2_ of isogenic strains in A^−^ backgrounds; scale bar: 2.5 mm. Parent strain DK1622 Δ*cglC*.

The *M. xanthus* genome contains four PilT paralogs, where *MXAN_6705* and *MXAN_6706* were highly conserved across Myxococcales (Fig. 2A). Phylogenetic analysis showed that PilT and PilU cluster together relative to other hexameric ATPases, indicating a close evolutionary relationship (Fig. 2B). Furthermore, PilU formed a clade with PilU proteins from other bacterial species, further supporting its annotation as PilU (Fig. S3A). Despite differences in sequence conservation, all five paralogs contain the hallmark Walker A and Walker B motifs, as well as the conserved Asp and His boxes characteristic of functional P-loop NTPases (Fig. 2C; Fig. S3B).

To investigate the roles of these PilT-like ATPases in S-motility, we generated in-frame deletion mutants in each of the three additional paralogs in an A⁻S^+^ strain. Unlike the Δ*pilU* mutant, these paralogs produced varying degrees of S-motility defects, with the Δ*MXAN_041*5 and Δ*MXAN_6706* eliciting the strongest phenotypes (Fig. 2D). Based on its phylogenetic relationship to PilT and its critical role in S-motility, further supports that *MXAN_1995* encodes a *bona fide pilU* gene.

### PilU is dispensable for pilus assembly and EPS production

**Fig 3:**
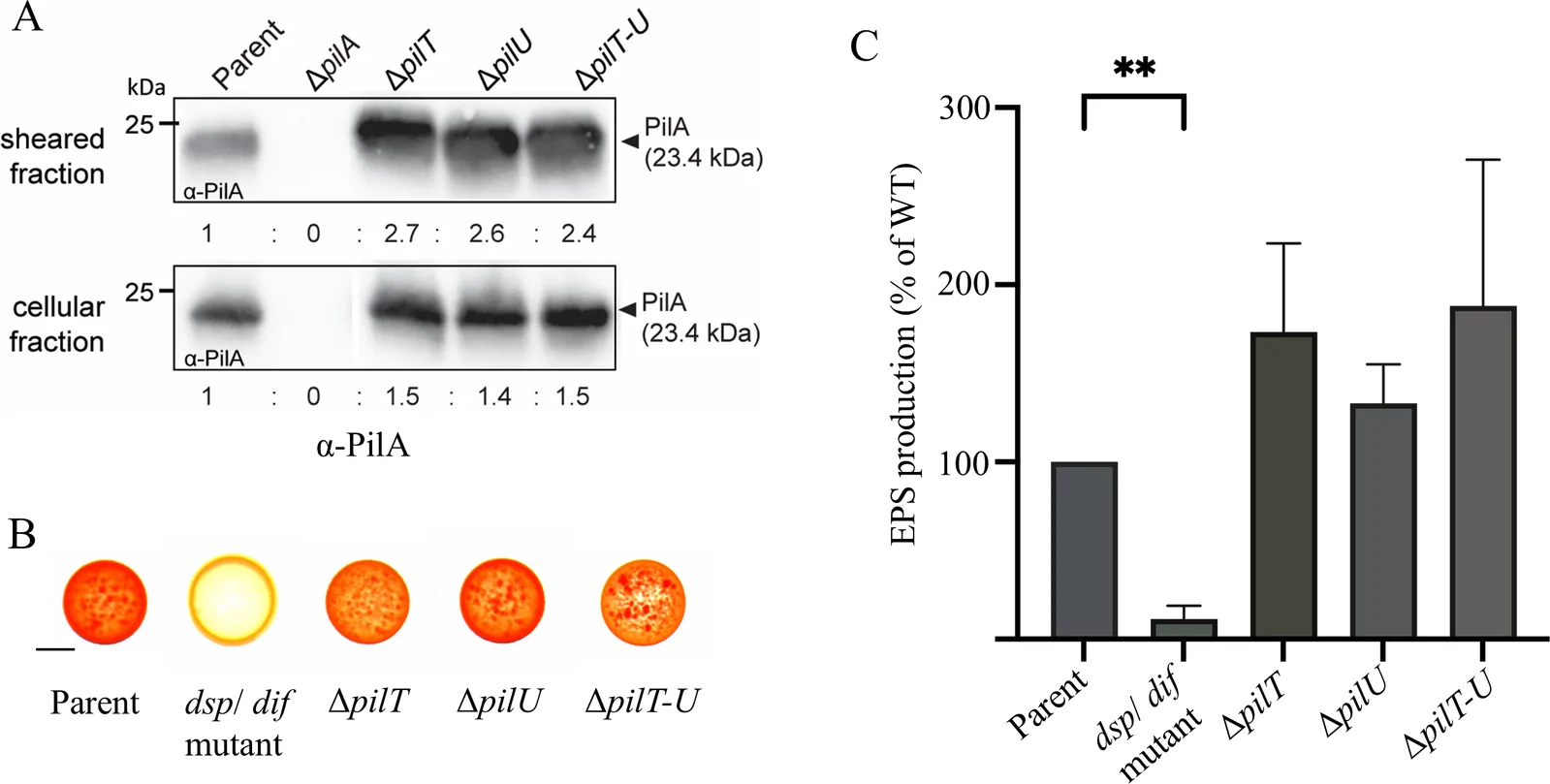
Pili and exopolysaccharide production in *pilT* and *pilU* mutants. (**A**) Shear-pili assay detecting PilA (24 kDa) by immunoblotting with polyclonal α-PilA antibodies. Bands were quantified and scored relative to the parent strain DK1217. (**B**) Congo red assay to assess EPS production; *dsp/dif* mutant serves as EPS-deficient control (yellow-orange color). Scale bar: 2.5 mm. (**C**) Quantitative trypan blue binding assay of EPS levels. Dye binding expressed as a percentage relative to the parent strain after subtraction of cell-free control. Error bars represent the mean and standard deviation from three biological replicates. Data were analyzed via two-tailed one-sample *t*-test. The double asterisk (**) denotes statistically significant differences in distributions (*P* < 0.0024) between parent strain and *dsp*/*dif* mutant. All assays were performed in biological triplicates, and representative images are shown.

Since T4P serves as the molecular engine to drive S-motility, we tested whether the Δ*pilU* mutant produced pili. For this, shear assays were performed in which surface-exposed thin pili were broken off by shear forces, followed by fractionation and immunoblotting with α-PilA antibody. The PilU mutant exhibited about 2.5-fold higher surface-exposed PilA compared with the parent strain, indicating that pilus assembly remained intact (Fig. 3A). Similar results were observed with a *pilT* and a *pilT-U* double mutant (Fig. 3A). A *pilA* mutant served as a negative control, in which no sheared pili were detected.

Myxobacteria secrete EPS and fibril proteins, which together form an extracellular matrix used as an S-motility substrate. To check whether the PilU mutant makes EPS, we measured its production qualitatively and quantitatively with Congo red and trypan blue assays, respectively. After a 5-day incubation, the *pilT*, *pilU*, and *pilT-U* double mutants bound Congo red like the parent strain, as they produced bright red colonies. In contrast, a *dsp/dif* mutant defective in EPS production (14, 37) served as a negative control and produced a yellow-orange colony due to its low dye-binding capacity (Fig. 3B). To measure EPS levels, trypan blue binding assays were performed. The *pilU* mutant showed higher levels of EPS (120%) than the parent, as did the *pilT* mutant (170%) and a double *pilT-U* mutant (180%) (Fig. 3C). Together, these data show that the Δ*pilU* mutant produces T4P and EPS, supporting a role of PilU in pili retraction, consistent with prior reports (24, 27).

### PilT and PilU exhibit self-association

**Fig 4:**
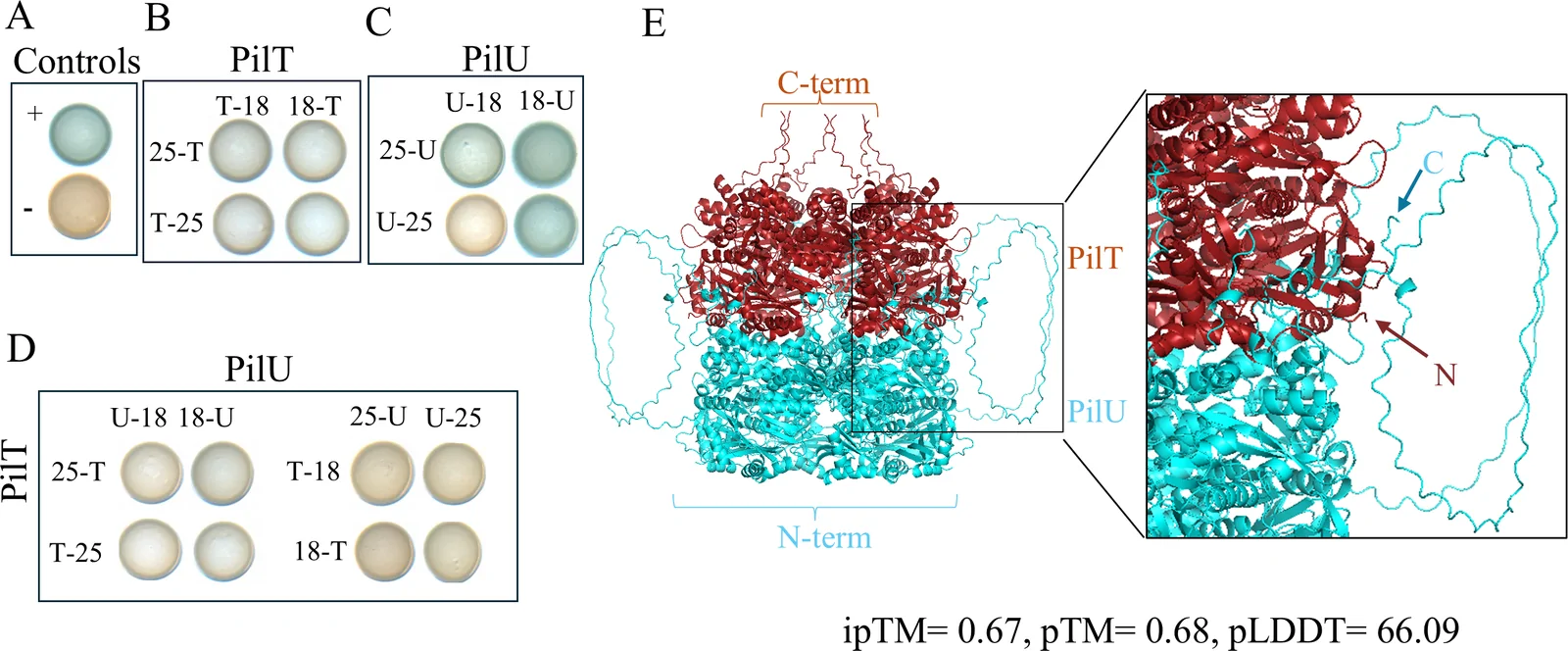
Assessment of PilT and PilU interactions and predicted homohexamer ring co-structures. BACTH assays testing pairwise interactions between T18- and T25-tagged PilT and PilU. (**A**) Leucine zipper fusions as a positive control and empty vectors as a negative control. (**B**) PilT self-interaction. (**C**) PilU self-interaction. (**D**) Absence of detectable interactions between PilT and PilU. (**E**) AlphaFold3-predicted co-structure of PilT (red) and PilU (cyan). Visible N- and C-termini are labeled. AlphaFold3 confidence scores for PilU homohexamer ipTM = 0.46, pTM = 0.5, pLDDT = 71.49; and PilT homohexamer, ipTM = 0.54, pTM = 0.59, pLDDT = 69.72.

To examine potential protein–protein interactions involving PilT and PilU, we employed the bacterial adenylate cyclase two-hybrid (BACTH) system, in which proteins of interest were co-expressed in an *E. coli cya* mutant as fusions to the T18 or T25 fragments of the *Bordetella pertussis* adenylate cyclase catalytic domain (38). Interactions between fusion partners reconstitute adenylate cyclase activity, leading to cAMP production and subsequent activation of the lactose or maltose operons. Protein–protein interactions were readily detected on indicator plates, where interacting fusion pairs produce blue-green colonies on X-gal plates, whereas non-interacting pairs yield colorless colonies (Fig. 4A).

Consistent with predicted hexameric architecture, BACTH analysis revealed strong self-interactions for both PilT and PilU (Fig. 4B and C). In contrast, no interaction was detected between PilT and PilU (Fig. 4D). Notably, AlphaFold predicted homo-oligomeric hexameric ring structures for PilT and PilU and, moreover, indicates a potential PilT–PilU association between their hexameric rings (Fig. 4E; Fig. S4A), where the N-terminal region of PilT binds toward the C-terminal region of the PilU hexamer that precedes the IDR, as similarly reported in *V. cholerae* (39). The absence of detectable interaction in the BACTH assay may suggest that assembled hexameric rings did not form in *E. coli* and were a prerequisite for PilT and PilU associations.

### PilU localizes to the cell poles independently of PilT

Many key components of the T4PM, including the extension motor PilB and the major retraction motor PilT, localize to the cell poles, and several of these proteins undergo dynamic pole-to-pole oscillations during cellular reversals. More precisely, the PilB extension ATPase localizes to the leading cell pole, while PilT is asymmetrically bipolarly localized (20).

**Fig 5:**
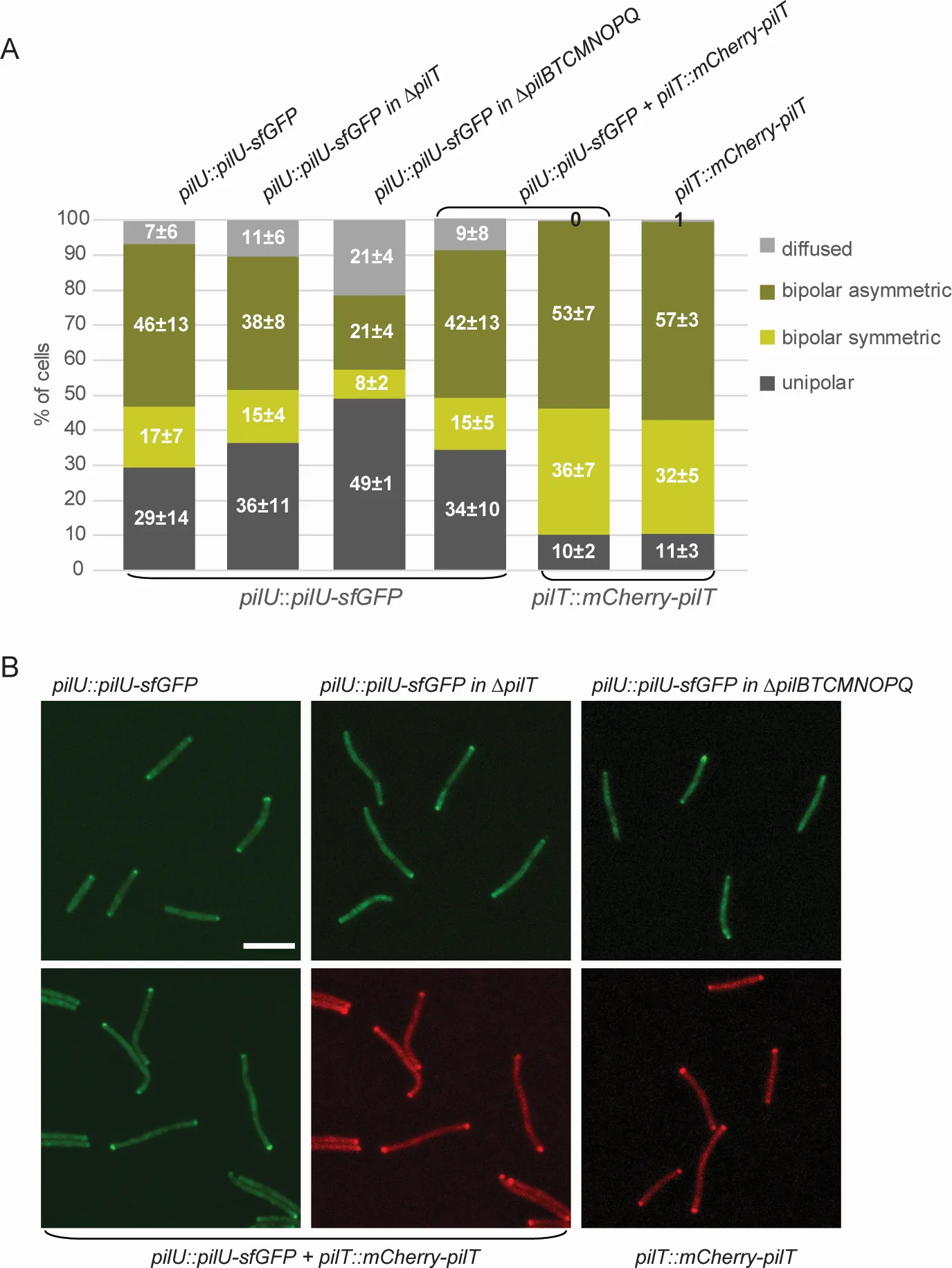
Polar localization of PilT and PilU. (**A**) Quantification of the polar localization of mCherry-PilT and PilU-sfGFP by fluorescence microscopy. Average percentage of cells in each localization pattern is shown with standard deviations from three independent experiments. (**B**) Representative micrographs of PilU-sfGFP and mCherry-PilT polar localization. Scale bar: 5 µm.

To determine the localization pattern of PilU, we constructed a C-terminal GFP fusion using a monomeric superfolder GFP variant expressed from its native locus. This PilU-sfGFP fusion was functional (Fig. S5A) and was stably expressed at the expected molecular mass (~80 kDa) (Fig. S5B). Fluorescence microscopy revealed that PilU-sfGFP showed predominantly bipolar localization (63% = 46% bipolar asymmetric + 17% bipolar symmetric), followed by unipolar localization (29%) and a low fraction of cells without polar signals (7%) (Fig. 5A and B). To compare the spatial distributions of the two retraction ATPases, we used a strain expressing an endogenous PilT-mCherry fusion. This reporter was functional (Fig. S5A) and stable (~87 kDa) (Fig. S5B). In comparison to PilU, PilT showed higher bipolar localization (89% = 57% bipolar asymmetric + 32% bipolar symmetric), decreased unipolar localization (11%), and almost no cells without polar signals (Fig. 5A and B). To assess their colocalization, we constructed a strain expressing endogenously expressed PilU-sfGFP and mCherry-PilT. In this strain, both PilT and PilU showed predominantly unipolar and bipolar asymmetric and symmetric localization (Fig. 5A and B; Fig. S5C and D). In the highest percentage of cells, PilU-sfGFP localized in a bipolar asymmetric pattern, whereas mCherry-PilT localized in a bipolar symmetric pattern (Fig. S5C and D). These localization distributions were similar whether PilT and PilU were expressed individually or simultaneously.

Together, these results demonstrate that PilT and PilU were enriched at one or both cell poles, supporting the idea that the two retraction ATPases were spatially coordinated during T4P function. To determine whether PilU localization depends on PilT as reported in *V. cholerae* (39), we analyzed the localization of PilU-sfGFP in the absence of *pilT*. However, we only found minor changes to PilU-sfGFP localization (Fig. 5A and B), indicating that PilU localization was independent of PilT and that it was recruited to the cell poles by a distinct targeting mechanism. In contrast, expressing PilU-sfGFP in a strain lacking the core T4P assembly components (Δ*pilBTCMNOPQ*) produced a 3-fold increase in diffuse cytoplasmic localization, a ~2-fold increase in unipolar localization, and a concomitant reduction in bipolar localization (Fig. 5A and B). These shifts suggest that although PilT was not required for PilU polar targeting, components of the core pilus assembly machinery or the extended T4P machine contribute to the stable polar enrichment of PilU. Finally, we note that overexpression of *pilU* in a *pilT-U* double mutant failed to restore motility, further demonstrating the central role PilT plays in pili retraction and motility (Fig. S6).

### PilU contributes to efficient single-cell T4P–dependent motility

**Fig 6:**
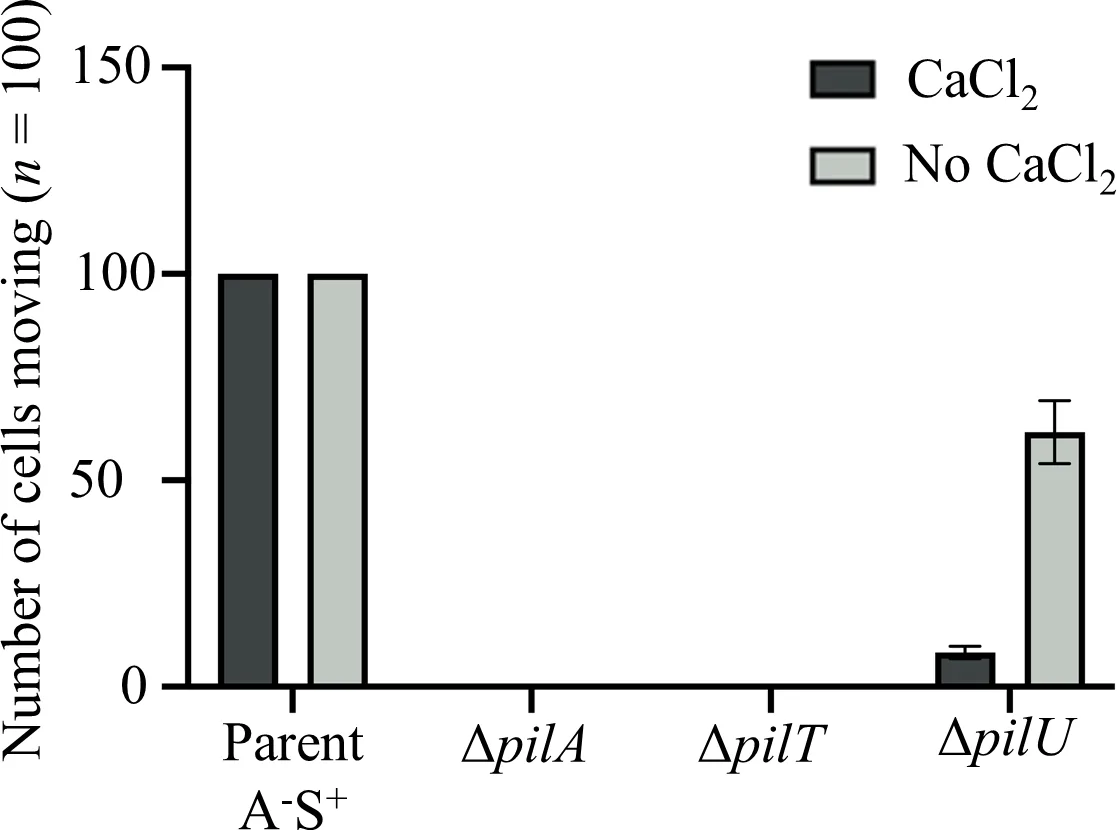
Calcium modulates *pilU*-independent motility. Single-cell motility of the parent strain and *pil* mutants in the presence and absence of 2 mM Ca²^+^. Time-lapse microscopy was performed over 30 min, with images acquired every 16 s. For each strain, 100 individual cells were tracked. Bar graph shows the number of cells that move at least once over a 30-min duration. Bars and error bars represent the mean and standard deviation from three biological replicates.

To assess the contribution of PilT and PilU to single-cell motility, we conducted time-lapse microscopy in strains that lacked A-motility (Fig. 6; Movies S1 to S4). For this, strains were grown to mid-log phase and spotted on a 0.5% agar pad in 0.5% CTT supplemented with 2 mM CaCl_2_, then incubated at 33°C for 1 h in a humid chamber, after which 100 individual cells were tracked over a 30-min period. As expected, the control cells (DK1217) displayed robust motility, with nearly all tracked cells exhibiting persistent movement during the image period. In contrast, the *pilT* mutant was completely non-motile, consistent with the established role of PilT as the primary T4P retraction motor. The Δ*pilU* mutant resulted in a strong but incomplete motility defect. While the majority of Δ*pilU* cells remained immobile, a small subpopulation (~10%) displayed detectable movement during the 30-min observation window. This residual motility was infrequent and characterized by short-lived movements with minimal net displacement, consistent with no net migration of *pilU* mutants on soft agar swarm plates.

### Calcium differentially modulates PilU-dependent motility

Calcium is a key divalent ion that modulates *M. xanthus* motility and development. Previous studies demonstrated that increasing extracellular Ca²^+^ enhances S-motility, influences the relative contributions of A- and S-motility, and promotes T4P-dependent behaviors (40–43). Specifically, calcium binding stabilizes PilY1.1 and associated minor pilin complexes, suggesting that Ca²^+^ directly affects T4P function. To address this, we examined whether PilU contributes to calcium-dependent regulation of motility. To do so, colony swarming of WT, Δ*pilT*, Δ*pilU*, and Δ*pilA* strains was examined on CTT soft agar in the presence and absence of CaCl₂ in both A^+^ and A^−^ backgrounds. In an A^+^ background in the absence of CaCl₂, the WT strain exhibited large irregular S-motility flares, while the Δ*pilU* mutant showed limited swarming accompanied by short, irregular flares at the colony edge (Fig. S7A). In the presence of CaCl₂, WT motility was enhanced in that colony expansion was evenly dispersed, while the Δ*pilU* colony showed reduced expansion and was also more evenly dispersed compared to the absence of CaCl₂ (Fig. S7A).

In A^−^ backgrounds in the absence of CaCl₂, the parent A^−^S^+^ strain exhibited irregular S-motility flares with moderate colony expansion, while the presence of calcium resulted in enhanced and evenly dispersed colony morphology (Fig. S7B). In contrast, in the presence of calcium, the swarming phenotype of the Δ*pilU* mutant was completely abolished (Fig. S7B). Given the opposite effects of CaCl₂ on the parent and Δ*pilU* mutant phenotypes, we next examined calcium-dependent effects on motility at the single-cell level in an A^−^ background. For the parent strain, time-lapse microscopy revealed that CaCl₂ did not change the number of cells moving (Fig. 6; Movie S5; compare to Movie S1). In contrast, Δ*pilU* cells exhibited very limited movement in the presence of CaCl₂, with only a small fraction of cells showing short, infrequent displacements. However, in the absence of CaCl₂, motility of the Δ*pilU* mutant was substantially increased, with approximately ~55% of cells displaying detectable movements that were more frequent and sustained than under calcium-supplemented conditions (Fig. 6; Movie S4; compare to Movie S8). For *pilA* and *pilT* mutants, there were no cell movements in the presence or absence of CaCl_2_ (Fig. 6; Movies S6 and S7; compared to Movies S2 and S3). Together, these results indicate that calcium differentially modulates T4P-driven motility of *pilU* mutants in the absence or presence of the A-motility gliding machinery, revealing an important role of PilU in supporting S-motility under calcium-rich conditions.

### Intragenic suppressor suggests a regulatory role for the PilU C-terminus

**Fig 7:**
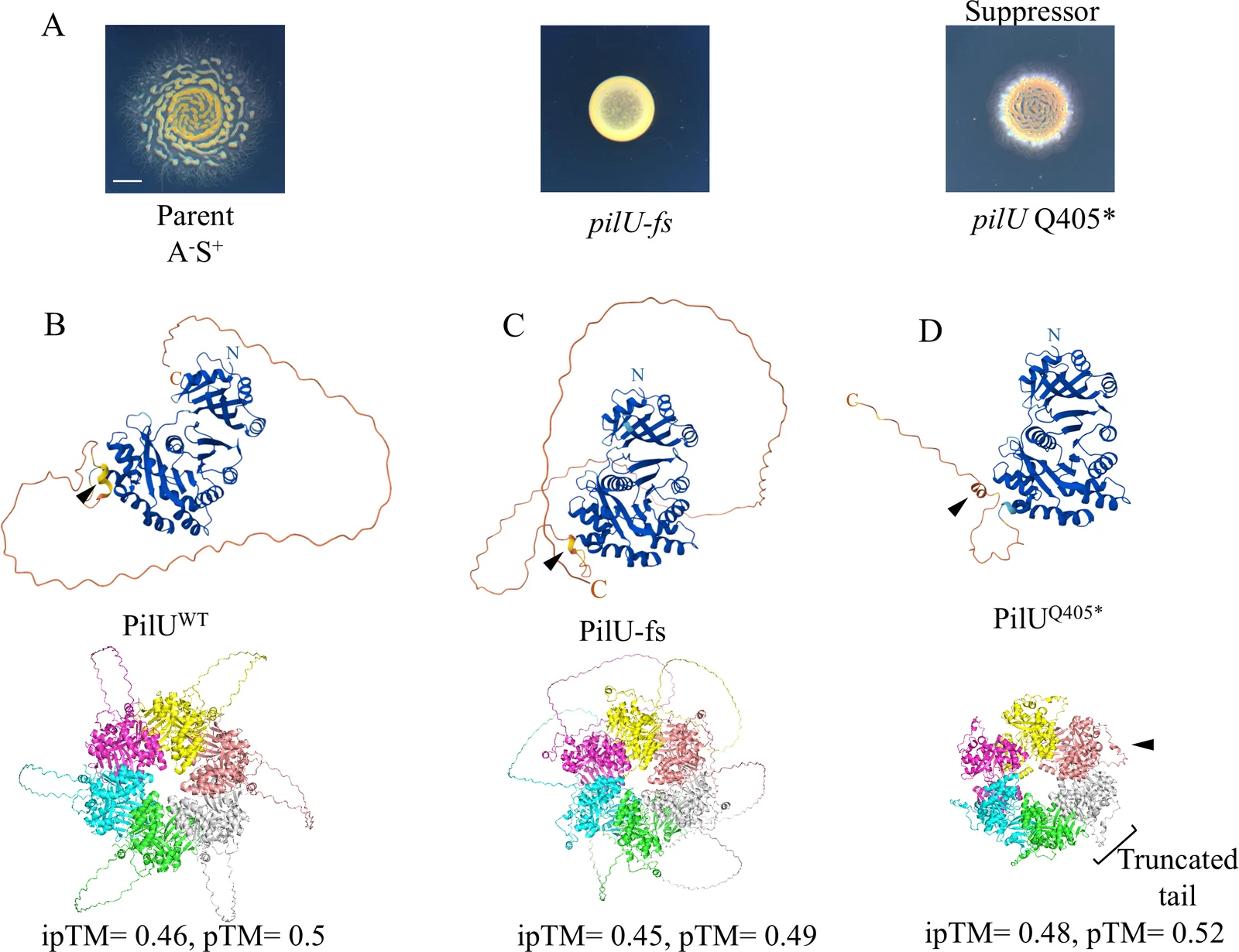
Intragenic suppression of the *pilU-fs* 2120 allele. (**A**) Colony phenotypes of the DK2120 *pilU-fs* mutant and an intragenic suppressor carrying a nonsense mutation (Q405*). Scale bar: 2.5 mm. Parent strain DK1218. (**B–D**) AlphaFold models of the PilU monomer (upper) and hexamer (lower) for WT PilU (**B**), PilU-fs mutant with a 36 amino acid extended C-terminus (**C**), and the Q405* intragenic suppressor with a truncated C-terminus (**D**). For reference, black triangles mark the short helix near the C-terminus.

During our studies, we found a spontaneous suppressor of the *pilU-fs* mutant that restored motility. The genome of this suppressor was sequenced and revealed an intragenic nonsense mutation near the 3′ end of the *pilU-fs* mutation. This mutation, Q405→NS, truncated PilU from its full length of 468 amino acids to 404 amino acids. Backcrossing this allele into the original *pilU-fs* background restored motility, demonstrating that this nonsense mutation conferred suppression (Fig. 7A). Structural predictions with AlphaFold indicated that PilU contains an apparent flexible, intrinsically disordered C-terminus (Fig. 7B; Fig. S4A). The DK2120 *pilU-fs* mutation at codon 454 extended the C-terminus by 36 amino acids (Fig. 7C), whereas the suppressor mutation shortened the WT protein by 64 amino acids and essentially removed the predicted C-terminal IDR (Fig. 7D; Fig. S4B). These findings suggest that the frameshift mutation, which elongates the C-terminus, interferes with PilU function, possibly by destabilizing PilU or by imposing a constitutive inhibitory effect.

## DISCUSSION

T4P-mediated motility in *M. xanthus* relies on tightly coordinated cycles of pilus extension, adhesion, and ATP-driven retraction. In this study, we identify PilU as an important yet previously unrecognized component of the T4P retraction machinery, along with PilT to power S-motility. Comparative genomics revealed that although *M. xanthus* encodes four PilT-like ATPases, deletion of *pilU* alone eliminates S-motility. Two of the remaining paralogs, *MXAN_0415* and *MXAN_6706*, exhibit partial S-motility defects, indicating that they too could serve auxiliary and perhaps conditional roles in pili function/retractions. Current investigations are testing these possibilities. Despite PilU’s lower occurrence among myxobacteria species, orthologs are broadly found in other diverse T4P systems. Notably, *pilU* resides within a conserved gene cluster and, in related genera such as *Archangium* and *Melittangium*, co-occurs with *pilY1* and minor pilin genes. These associated PilY1 proteins share sequence identity with the *M. xanthus* PilY1.3 variant (9), suggesting that *pilU* may have coevolved with specific minor pilin–PilY1 modules to fine-tune T4P-dependent motility.

Phenotypically, deletion of *pilU* closely resembles *pilT* loss-of-function at the colony level, producing smooth-edged, non-expanding colonies despite pilus production. However, unlike *pilT* mutants, which are immotile as single cells, *pilU* mutants retain limited surface motility. These movements are rare, transient, and result in negligible net displacement. Prior *M. xanthus* findings showed that retraction forces can be generated in the absence of PilT (3); however, the frequency and coordination of these retraction events are insufficient to drive motility. Additionally*,* PilT-independent retraction events occur at high velocity but low force (3), which likely explains the weak and non-productive movements observed in *pilU* mutants, consistent with insufficient force generation despite rapid retraction dynamics. Thus, the residual motility of *pilU* mutants further indicates that PilU is not the primary retraction motor but instead enhances or modulates PilT-dependent retraction, consistent with observations in *P. aeruginosa*, *V. cholerae*, and *Neisseria* spp. (24–26), where PilU functions as an accessory retraction ATPase to PilT. Furthermore, recent findings in *V. cholerae* found that PilT, and not PilU, binds the T4P core assembly machinery to engage PilC for pilus retraction (39). However, in this regard, *M. xanthus* appears to be different, where PilU and/or another PilT paralogs can engage PilC since pilus retraction still occurs in the absence of PilT (3). Additionally, unlike *pilT* null mutants, Δ*pilU* mutants shown here have conditional phenotypes where low temperatures or the absence of calcium partially restore S-motility. Our prior studies with *sglS* and *sglT* null mutants also exhibit Ts^−^ S-motility phenotypes (31, 32), suggesting that these two proteins may function in a PilU pathway. Finally, the inability of PilU overexpression to restore motility in a *pilT pilU* double mutant further supports the central role PilT plays in motility.

Importantly, the *pilU* deletion does not disrupt pilus assembly or EPS production, confirming that the S-motility defect is not the consequence of defective pilus or matrix assembly. Both *pilT* and *pilU* mutants displayed increased levels of surface-exposed pili relative to the WT, while EPS production was not significantly different from WT, suggesting that the motility defects were the result of impaired pilus retraction rather than altered EPS levels.

Our protein–protein interaction studies further clarify the relationship between PilT and PilU. Both ATPases exhibited self-interaction in the bacterial two-hybrid assay, consistent with the conserved hexameric architecture of T4P retraction motors. However, we did not detect a direct PilT–PilU interaction in a heterologous host, suggesting that monomers of these proteins do not interact. Experimental findings from other gram-negative bacteria found that PilT and PilU interact or form mixed assemblies (17, 24, 25, 44, 45). In *V. cholerae*, structural modeling, AlphaFold predictions, cryo-EM, and molecular dynamics simulations have suggested that PilT and PilU homohexamers assemble into coordinated complexes and that interactions between PilT N-terminus and PilU C-terminus are important for motor coordination during T4P retraction (39, 46, 47). Our AlphaFold studies similarly predict that the N-terminus of PilT hexamer rings interacts with the C-terminus of PilU hexameric rings to presumably provide full retraction force in *M. xanthus*.

**Fig 8:**
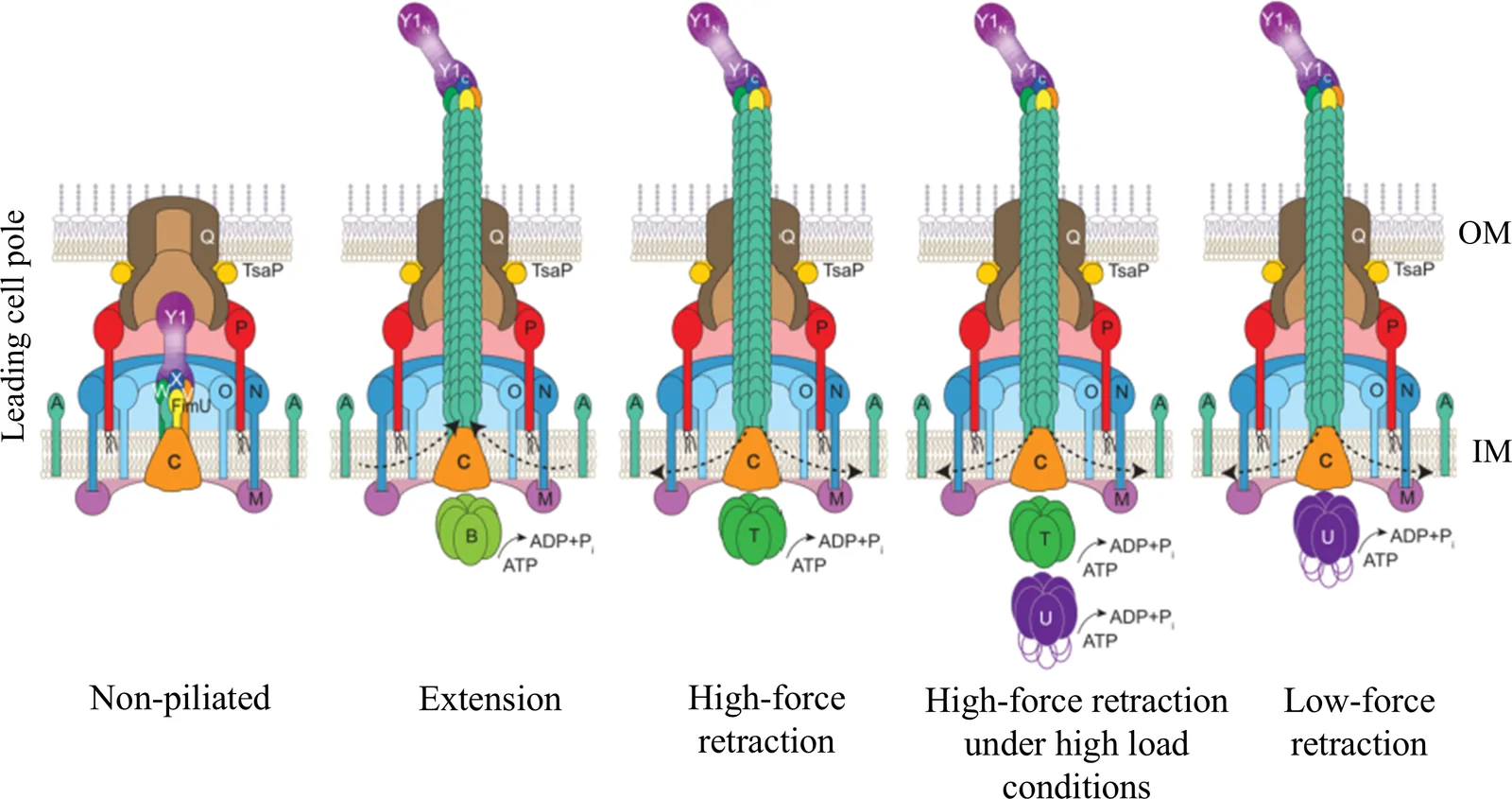
Model of T4PM at the leading cell pole in non-piliated and piliated states. At the leading cell pole, the T4PM can be unpiliated (left) or upon binding of PilB at the T4PM base the pilus polymerizes (2nd left). For retractions, PilT replaces PilB and drives depolymerization of the pilus fiber into the cytoplasmic membrane (middle). Under high-load conditions, PilU acts as an accessory retraction motor that together with PilT supports T4P retraction (2nd right). In absence of PilT, high velocity but low-force retraction can occur, presumably by PilU (right). Dashed arrows indicate incorporation or removal of the major pilin PilA at the pilus base during extension and retraction, respectively. Single-letters denote canonical Pil-prefix designations of each protein. Y1_N_ and Y1_C_ denote the N- and C-terminal domains of PilY1. Modified model derived from (9).

Spatial localization of the motors provides additional insight into their functional relationship. In *M. xanthus*, the extension ATPase PilB localizes predominantly to the leading pole, whereas the retraction ATPase PilT is enriched asymmetrically at both poles (20). When cells change their direction of movement, T4P assembles at the new leading pole, and PilB switches polarity. In contrast, studies in *P. aeruginosa* have shown that PilB and PilT can localize to both poles, while PilU is preferentially associated with the piliated pole (48). In our study, PilU–sfGFP localized primarily to one or both poles, closely mirroring the distribution pattern of PilT. Notably, PilU remained polar, even in the absence of PilT, indicating that its localization does not depend on PilT. It is tempting to speculate that SofG and/or BacP are involved in the polar localization of PilU, as has already been demonstrated for PilB and PilT (49). Frequent colocalization between PilT and PilU supports a model in which the two motors coordinate at the T4P base without necessarily forming stable hetero-hexamer complexes. The T4P machine at the leading pole can switch between an unpiliated state and an extension mode driven by PilB, while retraction is initiated when PilT replaces PilB to drive pilus depolymerization. We propose that, under high load, PilU acts with PilT to support high-force retraction, and that in the absence of PilT, PilU may mediate low-force retraction (Fig. 8).

We identified a spontaneous intragenic suppressor that restores motility to the *pilU* frameshift mutant. The suppressor introduces a nonsense mutation that truncates the C-terminus, including the 36-amino-acid extension generated by the 2120 frameshift mutation. The relatively long PilU extended C-terminal tail is unique to myxobacteria, where AlphaFold modeling suggests that it is intrinsically disordered. The frameshift mutation further elongates it, which may in turn disrupt hexamer stability or activate an auto-inhibition mechanism. Thus, the suppressor may relieve this interference, restoring functional hexamer formation or alleviating a negative regulatory function. Consistent with the latter, IDRs are frequently involved in protein–protein interactions (50, 51); thus, another factor may bind the PilU IDR to regulate its function, where SglS or SglT could play such a role. Another possible interpretation is that the original *pilU-fs* mutant resulted in an unstable protein that was subsequently stabilized by the intragenic suppressor. Regardless, these data underscore a role of the PilU C-terminus for WT motor activity, consistent with emerging evidence that flexible terminal regions of PilT-like ATPases modulate oligomer dynamics and ATPase cycling (15, 18, 46, 47).

Our data also reveal an unexpected calcium-dependent requirement for PilU. Whereas calcium enhances motility in WT cells, it abolishes the limited S-motility found in Δ*pilU* mutants. Calcium-dependent stabilization of adhesins and extracellular matrix components has been reported in other bacteria (52, 53), where it promotes surface attachment and early biofilm formation. Calcium binding also stabilizes PilY1 and ultimately allows minor pilin cluster to support T4P function under a broader range of environmental conditions (40, 41, 54). Moreover, *M. xanthus* cells dynamically adjust the relative contributions of A- and S-motility in response to calcium, shifting from predominantly A-motility at low calcium to enhanced S-motility at higher calcium concentrations (42). Our findings may reflect calcium-induced strengthening of pilus–EPS interactions that elevates mechanical load, rendering PilT alone insufficient for productive retraction and cell movement. Alternatively, calcium may differentially modulate PilT and PilU activities or their integration with downstream signaling systems. Together, these observations suggest that extracellular calcium regulates the PilT–PilU retraction system, allowing environmental cues to fine-tune T4P-dependent motility by modulating retraction force and its coordination.

Collectively, our results establish PilU as a functional PilT paralog and an accessory retraction motor that, together with PilT, supports high-force T4P retraction. This dual-motor system in *M. xanthus* reveals additional complexity in T4P regulation and suggests a mechanism for environmental control of retraction dynamics. Future studies integrating biophysics, structural analysis, and high-resolution imaging will help clarify how PilT and PilU coordinate force generation to drive multicellular behavior.

## MATERIALS AND METHODS

### Bacterial strains and growth conditions

All bacterial strains used in this study are listed in Table S1. *M. xanthus* cultures were grown in CTT medium (1% [wt/vol] casitone, 10 mM Tris-HCl pH 7.6, 1 mM KH₂PO₄, 8 mM MgSO₄) at 33°C in the dark with shaking. *E. coli* strains were maintained in LB medium. When required, antibiotics were added at the following final concentrations: kanamycin (50 µg/mL), oxytetracycline (10 µg/mL), or ampicillin (100 µg/mL). Cells were washed in TPM buffer (CTT lacking casitone). Solid media were prepared by adding agar to 1.5% or 0.5%.

### Sequencing and genetic mapping

Genomic DNA from DK2120 was extracted and sequenced using an Illumina NextSeq 2000 platform (MiGS, Pittsburgh, PA), generating paired-end reads (2 × 151 bp). Reads were mapped to the *M. xanthus* DK1622 reference genome using BreSeq version 0.38.1 with default parameters. Candidate mutations were identified from resulting variant calls and confirmed by PCR and Sanger sequencing.

### Plasmid and strain construction

Plasmids and primers are listed in Tables S2 and S3. For rescue experiments, the full-length *MXAN_1995* WT gene was amplified using Q5 high-fidelity DNA polymerase (NEB) and cloned into pMR3487 (55). Constructs were propagated in *E. coli* DH5α, verified by colony PCR, restriction digestion, and sequencing, and then electroporated into *M. xanthus* for chromosomal integration via homologous recombination under oxytetracycline selection.

Markerless deletions, as well as integration of *pilU-sfGFP* and *mCherry-pilT,* were generated using a two-step homologous recombination strategy (56). Verified constructs were electroporated into *M. xanthus*, selected on kanamycin, and then counter-selected for plasmid excision on 2% galactose–CTT agar (57). Successful deletions and insertions were confirmed by PCR using primers flanking the deletion/insertion site and phenotypic analysis.

### Motility assays

*M. xanthus* cultures were grown in CTT to mid-log phase, washed with TPM buffer, and normalized to 1.5  ×  10⁹ cfu/mL. To assess S-motility, 5 µL of culture was spotted onto 0.5% agar CTT plates supplemented with 2 mM CaCl₂ (unless stated otherwise). Plates were incubated at 33°C and imaged after 72 h using an Olympus SZX10 stereoscope.

For single-cell movement analysis, cells were spotted on 0.5% agar CTT glass slides containing 2 mM CaCl₂ (unless stated otherwise) and incubated at 33°C for 1 h in a humid chamber. Time-lapse imaging was performed using an Olympus IX83 inverted microscope equipped with a 60 × oil immersion objective and an Orca-Flash4.0 LT sCMOS camera.

### EPS assays

EPS production was examined by spotting 5 µL of cell suspensions (3  ×  10⁸ cfu/mL in TPM) onto CTT plates containing 30 µg/mL Congo red. Plates were incubated at 33°C for 5 days before imaging (31). Retention of trypan blue was carried out as previously described (58). Cells from overnight cultures were resuspended in TPM to OD₆₀₀  =  1.0. A 900 µL aliquot was mixed with 100 µL of trypan blue (100 mg/mL). Samples were incubated for 1 h at room temperature with gentle rocking and then centrifuged at 16,000  ×  *g* for 5 min. The top 900 µL of supernatant was transferred to cuvettes, and absorbance at 585 nm was measured. Values were normalized to WT levels.

### Fluorescence microscopy and image analysis

Fluorescence microscopy was performed as described earlier (20). Briefly, exponentially growing cells were placed on a coverslip and overlaid with an agarose pad (1% SeaKem LE agarose [Cambrex] with TPM buffer [10 mM Tris-HCl pH 7.6, 1 mM KH_2_PO_4_ pH 7.6, 8 mM MgSO_4_] and 0.2% CTT). After 30 min at 32°C, cells were visualized using a temperature-controlled Leica DMi8 Thunder imager inverted microscope, and phase contrast and fluorescence images were acquired using a Leica K8 CMOS camera and the LASX software (Leica Microsystems). Microscope images were processed with Fiji (59), and cell masks were determined using Oufti (60) and manually corrected when necessary. To precisely quantify the localization of fluorescently labeled proteins, we used Matlab R2020a (The MathWorks) in an established analysis pipeline (61). For each cell with polar cluster(s), an asymmetry index (ꞷ) was calculated as (ꞷ = (total fluorescence at pole 1 – total fluorescence at pole 2) / (total fluorescence at pole 1 + total fluorescence at pole 2), with pole 1 always being the pole with the highest fluorescence. The localization patterns are categorized as follows: unipolar (ꞷ > 0.9), bipolar asymmetric (0.9 ≥ ꞷ ≤ 0.2), and bipolar symmetric (ꞷ < 0.2). The quantifications shown are averaged data from three independent experiments. For each strain in every triplicate, at least 300–900 single cells were analyzed.

### Pili shear assay

Pili were sheared from *M. xanthus* cells using an established protocol (20). Briefly, pili were isolated from *M. xanthus* cells grown on 1% CTT/1.5% agar for 2-3 days. All strains have the DK1217 parent background, except the negative control strain SA7705, which is ∆*pilA∆aglQ*. Cells were scraped and resuspended in pili resuspension buffer (100 mM Tris-HCl pH 7.6, 150 mM NaCl; 1 mL per 60 mg cells). Suspensions were vortexed at maximum speed for 10 min. A 100 µL aliquot was collected for whole-cell lysates. The remaining suspension was centrifuged at 13,000  ×  *g* at 4°C, and supernatants were clarified by two additional rounds of centrifugation. Pili were precipitated using 10× pili precipitation buffer (final concentrations: 100 mM MgCl_2_, 500 mM NaCl, and 2% PEG 6000) overnight at 4°C and then resuspended in SDS lysis buffer for SDS-PAGE. The pili shear data shown were reproduced in two additional independent experiments with similar results.

### Immunoblotting

Whole-cell fractions or isolated pili were separated by SDS-PAGE. Rabbit primary antibodies included α-PilA, α-mCherry, and α-GFP. HRP-conjugated goat anti-rabbit secondary antibody (1:15,000) was used for detection. Blots were developed using HRP substrate (Millipore) and imaged with a Bio-Rad ChemiDoc MP image analyzer.

### BACTH assays

BACTH assays were performed according to the manufacturer’s protocol (Euromedex) (38). Briefly, plasmids encoding full-length PilT and PilU fused N-terminally or C-terminally to the T25 or T18 *B. pertussis* adenylate cyclase (CyaA) fragments were transformed into *E. coli* BTH101 alone or in pairs. As a positive control, BTH101 co-transformed with the plasmids pKT25-zip and pUT18C-zip. Transformed cells were incubated at 30°C for 24 h, outgrown with 500 μL LB for 1 h at 37°C with shaking. Transformants were isolated on LB plates containing kanamycin, 50 μg/mL, and ampicillin, 100 μg/mL, to select for both plasmids. Strains were then grown statically at 30°C from frozen stocks overnight in LB supplemented with kanamycin and ampicillin, and 3 μL of each culture was then spotted onto LB plates containing kanamycin, ampicillin, 0.5 mM IPTG, and 40 μg/mL X-gal, and incubated at 30°C for 48 h.

### AlphaFold-multimer modeling

The PilU structural model was generated using AlphaFold 3 (62). PyMOL v2.4.1 (Schrödinger LLC) was used for visualization and analysis.

### Bioinformatics analyses

Protein co-occurrence with PilU was examined using webFlaGs (63). Sequence alignments were produced with Clustal Omega with default parameters (64). Phylogenetic trees were generated in MEGA12 using the maximum likelihood method with Poisson correction and 100 bootstrap replicates (65).

## SUPPLEMENTAL MATERIAL

Supplemental material jb.00248-26-s0001.docx Table S1 to S3; Fig. S1 to S6.

Movie S1 jb.00248-26-s0002.avi Time-lapse microscopy of S-motility in parent strain (DK1217) that lacks A-motility. Video made on 0.5% agar pad in 0.5% CTT with 2 mM CaCl₂ and spans 30 min with 16 s frame intervals. Scale bar: 20 µm.

Movie S2 jb.00248-26-s0003.avi Time-lapse microscopy of Δ*pilA* mutant as described for Movie S1.

Movie S3 jb.00248-26-s0004.avi Time-lapse microscopy of Δ*pilT* mutant as described for Movie S1.

Movie S4 jb.00248-26-s0005.avi Time-lapse microscopy of Δ*pilU* mutant as described for Movie S1.

Movie S5 jb.00248-26-s0006.avi Time-lapse microscopy of S-motility in parent strain (DK1217) that lacks A-motility. Video made on the same condition as described for Movie S1 but without CaCl2.

Movie S6 jb.00248-26-s0007.avi Time-lapse microscopy of Δ*pilA* mutant as described for Movie S5.

Movie S7 jb.00248-26-s0008.avi Time-lapse microscopy of Δ*pilT* mutant as described for Movie S5.

Movie S8 jb.00248-26-s0009.avi Time-lapse microscopy of Δ*pilU* mutant as described for Movie S5.

ASM does not own the copyrights to Supplemental Material that may be linked to, or accessed through, an article. The authors have granted ASM a non-exclusive, world-wide license to publish the Supplemental Material files. Please contact the corresponding author directly for reuse.
