# Supplementary material for: Discovery of PilU as a second type IV pilus retraction motor in Myxococcus xanthus
Source: J Bacteriol. 2026 Aug 12;208(9):e00248-26. doi: 10.1128/jb.00248-26 (PMC13584747; doi:10.1128/jb.00248-26)
Supplement: Supplementary file 1 [file jb.00248-26-s0001.docx]

**Supplementary Material**

**Discovery of PilU as a second Type IV pilus retraction motor in *Myxococcus xanthus***

Mahdia Rahman^a^, Kalpana Subedi^a^, Andrea Harms^b^, Daniel Wall^a^, and Anke Treuner-Lange^b^

^a^Department of Molecular Biology, University of Wyoming, Laramie, Wyoming, USA

^b^Max Planck Institute for Terrestrial Microbiology, Germany

**Table of Contents**

**Tables S1-S3**

**Figures S1-S6**

**Supplemental videos 1-8**

**References**

**Table S1** Strains used in this study

| **Strain** | **Genotype** | **Source or reference** |
| --- | --- | --- |
| DH5α | *E. coli* cloning strain | Lab collection |
| DK1218 | A^–^ S^+^, *cglB2* | [1] |
| DK2120 | A^–^ S^+^, *cglB2* (*MXAN_1995* C1362) | [1] |
| DW3000 | A^–^ S^+^, *cglB2,* P*_IPTG_-MXAN_1995*^WT^ | This study |
| DK1622 | A^+^S^+^, WT *M. xanthus* | [2] |
| DK1217 | A^–^ S^+^, *aglB1* (*aglQ1,* F36→I) | [2] |
| DK8601 | A^–^ S^–^, Δ*pilA* *aglB1* | [3] |
| DK10410 | A^+^S^−^, Δ*pilA* | [1] |
| DK10409 | A^+^S^−^, Δ*pilT* | This study |
| DW3001 | A^–^ S^–^, Δ*pilT* *aglB1* | This study |
| DW3002 | DK1217 Δ*pilU* | This study |
| DW3003 | DK1622 Δ*pilU* | This study |
| DW3004 | DK1217 Δ*pilT* Δ*pilU* | This study |
| DW1490 | DK1622 Δ*cglC* | Lab collection |
| DW3005 | Δ*cglC* Δ*MXAN_6705* | This study |
| DW3006 | Δ*cglC* Δ*MXAN_6706* | This study |
| DW3007 | Δ*cglC* Δ*MXAN_0415* | This study |
| DW3008 | Δ*cglC* Δ*pilU* | This study |
| DW3009 | Δ*cglC* Δ*pilT* | This study |
| DK1219 | A^–^ S^+^, *cglC1* (52I →FS) | [1, 4] |
| DK1650 | *cglC1 dsp* | [1] |
| SA6024 | DK1622 Δ*pilBTCMNOPQ* | [5] |
| SA9307  SA7705 | DK1622 *pilT::mCherry-pilT*  DK1622 Δ*pilA* Δ*aglQ* | [6]  This study |
| TL99 | DK1622 *pilT::mCherry-pilT*, *pilU::pilU-sfGFP* | This study |
| TL100 | DK1622 *pilU::pilU-sfGFP* | This study |
| TL263  TL271 | DK1622 *pilU::pilU-sfGFP* Δ*pilT*  DK1622 *pilU::pilU-sfGFP* Δ*pilBTCMNOPQ* | This study  This study |
| DW3010 | DK2120 *pilU*^Q405*^ | This study |

**Table S2** Plasmids used in this study

| **Plasmid** | **Relevant features** | **Source or reference** |
| --- | --- | --- |
| pBJ113/114 | Km^R^, *galK*¸ vector for generating in-frame deletions | [7] |
| pMR3487 | IPTG-inducible promoter, Tc^R^ | [8] |
| pKT25 | Vector for C-terminal fusion of genes to the T25 fragment of the *Bordetella pertussis* adenylate cyclase gene; Km^R^ | Euromedex  (BACTH kit) |
| pKNT25 | Vector for N-terminal fusion of genes to the T25 fragment of the *B. pertussis* adenylate cyclase gene; Km^R^ | Euromedex  (BACTH kit) |
| pUT18 | Vector for N-terminal fusion of genes to the T18 fragment of the *B. pertussis* adenylate cyclase gene; Amp^R^ | Euromedex  (BACTH kit) |
| pUT18C | Vector for C-terminal fusion of genes to the T18 fragment of the *B. pertussis* adenylate cyclase gene; Amp^R^ | Euromedex  (BACTH kit) |
| pKS152 | P*_IPTG_-MXAN_1995*^WT^, Tc^R^ | This study |
| pMR01 | pBJ114; for generation of an in-frame deletion of *MXAN_1995* | This study |
| pMR02 | pBJ114; for generation of an in-frame deletion of *MXAN_5787* | This study |
| pDK23 | pBJ114; for the generation of an in-frame deletion of *cglC* | [9] |
| pSC58 | *pilT* in pKT25 | [10] |
| pSC60 | *pilT* in pKNT25 | [10] |
| pSC71 | *pilT* in pUT18 | [10] |
| pSC72 | *pilT* in pUT18C | [10] |
| pMR03 | *pilU* in pKT25 | This study |
| pMR04 | *pilU* in pKNT25 | This study |
| pMR05 | *pilU* in pUT18 | This study |
| pMR06 | *pilU* in pUT18C | This study |
| pMAT92 | pBJ114; for generation of an in-frame deletion of *MXAN_6705* | This study |
| pMAT93 | pBJ114; for generation of an in-frame deletion of *MXAN_6706* | This study |
| pIB132  pBJ∆aglQ | pBJ114; for generation of an in-frame deletion of *MXAN_0415*  pBJ114; for generation of an in-frame deletion of *MXAN_6861* | This study  [11] |
| pMEM33 | pBJ114; for integration of *mCherry-pilT* at native locus | [6] |
| pMAT594 | pBJ114; for integration of *pilU-msfGFP* at native locus | This study |

**Table S3** Primers used in this study

| **Primer name** | **Sequence (5’→3’)** | **Comments** |
| --- | --- | --- |
| pMR3487 *pilU*^WT^-F | TCTAGAAAATGGAACTCAACGAGATCC | pKS152 |
| pMR3487 *pilU*^WT^-R | GGTACCGAAGCGCTCGATCTG | pKS152 |
| *pilU*_del_Frag1_F | CAATTTCACACAGGAAACAGCTATGACCATGATTACGCC  AAGCTTATGCCCATCAACCAAGAGCTCG | pMR01 |
| *pilU*_del_Frag1_R | TAGAAGCGCTCGATCTGGAAGAGGATCTCGTTGAGTTCCATGAGC | pMR01 |
| *pilU*_del_Frag2_F | TGGAACTCAACGAGATCCTCTTCCAGATCGAGCGCTTCTAGC | pMR01 |
| *pilU*_del_Frag2_R | GGGTTTTCCCAGTCACGACGTTGTAAAACGACGGCCAGT  GAATTCCGGAGCCCACGAAGAAGG | pMR01 |
| *pilU*_del_F | GATCCTCAACCAGCCCCTC | pMR01 |
| *pilU*_del_R | GAGCGGAAGGTGCGTGAG | pMR01 |
| *pilT*_del_Frag1_F | CAATTTCACACAGGAAACAGCTATGACCATGATTACGCC  AAGCTTTTCGAGACGGCGGAAATCGGC | pMR02 |
| *pilT* _del_Frag1_R | GAACTAACGACCACCCGCTCCGAGCTGGTGCAGGTTGGCCAC | pMR02 |
| *pilT* _del_Frag2_F | GTGGCCAACCTGCACCAGCTCGGAGCGGGTGGTCGTTAGTTC | pMR02 |
| *pilT* _del_Frag2_R | GGGTTTTCCCAGTCACGACGTTGTAAAACGACGGCCAGT  GAATTCTTGAGGATGGCGTCGAGGATAC | pMR02 |
| *pilT* _del_Fwd | ATCAACGGCGCCTCCGCC | pMR02 |
| *pilT* _del_Rev | TTGACGGGGTTCAGCCCG | pMR02 |
| B2H-*Xba1*-PilU-F | ATGCAT TCTAGAGGAACTCAACGAGATCCTCC | BATCH |
| B2H-PilU-*Kpn1*-R | ATGCAT GGTACCGGGAAGCGCTCGATCTGGAAG | BACTH |
| *pilU*-sfGFP endo up+ EcoRI | gcgcgaattcccagcgcgaggtgggtgtggac | pMAT594 |
| *pilU*-sfGFP endo up - | catggagccgccgccgccgaagcgctcgatctg | pMAT594 |
| *pilU-nostop-sfGFP+* | cagatcgagcgcttcggcggcggcggctccatg | pMAT594 |
| *sfGFP-down-pilU-* | ggtgggcgccgcgaaccgctatttgtagagctc | pMAT594 |
| *sfGFP-down-pilU+* | gagctctacaaatagcggttcgcggcgcccacc | pMAT594 |
| *pilU-down-HindIII-* | gcgcaagcttccatgcaccccgtactcgcccgcttcc | pMAT594 |
| *o6705-A EcoRI* | atcgaattcgctcaagcccttcatcca | pMAT92 |
| *o6705-B NotI* | gcgcgcggccgccaacagttctgcgagcg | pMAT92 |
| *o6705-C NotI* | gcgcgcggccgcctcgctgatgggaagg | pMAT92 |
| *o6705-D PstI* | gcgcctgcagctcgcggcccgctgagcgg | pMAT92 |
| *o6706-A EcoRI* | gcgcgaattccagcgcgctcaagggcagcc | pMAT93 |
| *o6706-B NotI* | gctcgcggccgcggaccgccagacggggcgtc | pMAT93 |
| *o6706-C NotI* | gtccgcggccgcgagtccctgaagtcgctgggctag | pMAT93 |
| *o6706-D SalI* | gcaggtcgacgttctccaggtaggtgggggac | pMAT93 |
| *o0415-A-EcoRI* | atcgaattcgacggagcgcggctcacc | pIB132 |
| *o0415-B-NheI* | atcgctagccacaccgaccgcgagcag | pIB132 |
| *o0415-C-NheI* | atcgctagccagcgcgcgctcgagttc | pIB132 |
| *o0415-D-HindIII* | atcaagcttcgatgcccaacgtgggga | pIB132 |

Restriction sites underlined.

**Supplementary Fig. 1. Co‑occurrence of *pilU* and *pilY1* in Myxococcales.** Synteny analysis showing that *pilU* (black) co‑occurs with *pilY1* (purple) and pilus‑associated genes in selected Myxococcales species. In distant bacterial species the *pilU* and *pilT* genes are adjacent.

**Supplementary Fig. 2. Ts^−^ phenotype of *pilU* mutants.** Mid-log phase cultures of parent and mutant strains were spotted on 0.5% agar supplemented with 0.5% under indicated conditions. Images were taken after 72 hr. Scale bar, 2.5 mm.

A

B

**Supplementary Figure 3**. (A) Phylogeny of T4P motor ATPases from *M. xanthus* (MXAN), *P. aeruginosa* (PA), *V. cholerae* (VC), and *N. meningitidis* (NM). Tree constructed in MEGA12 using the Maximum Likelihood method based on amino acid sequences. Numbers at nodes are bootstrap support values (%) obtained from 100 replicates. (B) Alignment of *M. xanthus* PilT paralogs. Conserved Walker A, Asp, Walker B, and His boxes indicated. 2120 fs mutation marked with black triangle, whereas the intragenic nonsense Q405* suppressor mutation is marked with a red triangle.

**Supplementary Fig. 4. AlphaFold3 predictions of PilT–PilU and PilT–PilU^Q405*^ complexes.** Predicted structures for (A) the PilT–PilU complex and (B) the PilT–PilU^Q405*^ complex. Upper panels show predicted dimer-of-hexamers assemblies, whereas the lower panels show the corresponding predicted aligned error (PAE) maps and model confidence scores. The individual PilT and PilU hexamers were predicted with high confidence, and the inter-complex interfaces exhibited confidence metrics consistent with a potential direct interaction.

**Supplementary Fig. 5. Functionality and localization of PilU-sfGFP and mCherry-PilT fusions**. (A) Phenotype analysis of strains assayed on 0.5% agar supplemented with 1% CTT. Pictures taken at 24 hr. Scale bar, 1 mm. (B) PilU-sfGFP (80 kDa) and mCherry-PilT (87 kDa) expressed as full-length proteins. Total cell extracts of ~10^8^ cells separated by SDS-PAGE and probed with α-GFP antibodies (top), α-mCherry antibodies (middle) and following membrane stripping with α-LonD antibodies as loading controls (bottom). Reporters expressed from their native loci. (C) Distribution percentages of different colocalization patterns of PilU-sfGFP and mCherry-PilT in strain SA15363 from one experiment in which 510 single cells were analyzed. (D) Images of all cell colocalization patterns of PilU-sfGFP (top) and mCherry-PilT (bottom) in strain SA15363. Scale bar, 5 µm.

**Supplementary Fig. 6. PilU overexpression does not bypass PilT function.** PilU^WT^ expressed from an IPTG inducible promoter in a Δ*pilT* Δ*pilU* strain background and assessed for motility on 0.5% agar in 0.5% CTT with 2 mM CaCl_2_ at indicated IPTG concentration and imaged after 3 days, Scale bar, 2.5 mm.

**Supplementary Fig. 7. S-motility phenotypes of *pilT* and *pilU* mutants in A⁺ and A^ꟷ^ backgrounds.** Colony morphology of the parent and indicated mutants grown in the presence (2 mM) and absence of Ca²⁺ in the A**⁺** (A) and A^ꟷ^ (B) backgrounds. Scale bars, 2.5 mm.

**Supplementary Video 1.** Time-lapse microscopy of S-motility in parent strain (DK1217) that lacks A-motility. Video on 0.5% agar pad in 0.5% CTT with 2 mM CaCl_2_ and spans 30 minutes with 16 sec frame intervals. Scale bar, 20 µm.

**Supplementary Video. 2.** Time-lapse microscopy of Δ*pilA* mutant as described in Video S1.

**Supplementary Video. 3**. Time-lapse microscopy of Δ*pilT* mutant as described in Video S1.

**Supplementary Video. 4.** Time-lapse microscopy of Δ*pilU* mutant as described in Video S1.

**Supplementary Video. 5.** Time-lapse microscopy of S-motility in parent strain (DK1217) that lacks A-motility as made in Video S1 but without CaCl_2_.

**Supplementary Video. 6.** Time-lapse microscopy of Δ*pilA* mutant as described in Video S5.

**Supplementary Video. 7.** Time-lapse microscopy of Δ*pilT* mutant as described in Video S5.

**Supplementary Video. 8.** Time-lapse microscopy of Δ*pilU* mutant as described in Video S5.

**References**

1.	Wu, S.S., J. Wu, and D. Kaiser, *The Myxococcus xanthus pilT locus is required for social gliding motility although pili are still produced.* Mol Microbiol, 1997. **23**(1): p. 109–21.

2.	Dey, A., et al., *Sibling Rivalry in Myxococcus xanthus Is Mediated by Kin Recognition and a Polyploid Prophage.* J Bacteriol, 2016. **198**(6): p. 994–1004.

3.	Wall, D., S.S. Wu, and D. Kaiser, *Contact stimulation of Tgl and type IV pili in Myxococcus xanthus.* J Bacteriol, 1998. **180**(3): p. 759–61.

4.	Pathak, D.T. and D. Wall, *Identification of the cglC, cglD, cglE, and cglF genes and their role in cell contact-dependent gliding motility in Myxococcus xanthus.* J Bacteriol, 2012. **194**(8): p. 1940–9.

5.	Friedrich, C., I. Bulyha, and L. Sogaard-Andersen, *Outside-in assembly pathway of the type IV pilus system in Myxococcus xanthus.* J Bacteriol, 2014. **196**(2): p. 378–90.

6.	Oklitschek, M., et al., *Combinatorial control of type IVa pili formation by the four polarized regulators MglA, SgmX, FrzS, and SopA.* J Bacteriol, 2024. **206**(11): p. e0010824.

7.	Julien, B., A.D. Kaiser, and A. Garza, *Spatial control of cell differentiation in Myxococcus xanthus.* Proc Natl Acad Sci U S A, 2000. **97**(16): p. 9098–103.

8.	Iniesta, A.A., et al., *Two systems for conditional gene expression in Myxococcus xanthus inducible by isopropyl-beta-D-thiogalactopyranoside or vanillate.* J Bacteriol, 2012. **194**(21): p. 5875–85.

9.	Jakobczak, B., et al., *Contact- and Protein Transfer-Dependent Stimulation of Assembly of the Gliding Motility Machinery in Myxococcus xanthus.* PLoS Genet, 2015. **11**(7): p. e1005341.

10.	Potapova, A., L.A.M. Carreira, and L. Sogaard-Andersen, *The small GTPase MglA together with the TPR domain protein SgmX stimulates type IV pili formation in M. xanthus.* Proc Natl Acad Sci U S A, 2020. **117**(38): p. 23859–23868.

11.	Sun, M., et al., *Motor-driven intracellular transport powers bacterial gliding motility.* Proc Natl Acad Sci U S A, 2011. **108**(18): p. 7559–64.
